# Publisher Correction: Establishing magneto-structural relationships in the solid solutions of the skyrmion hosting family of materials: GaV_4_S_8−*y*_Se_*y*_

**DOI:** 10.1038/s41598-020-72919-2

**Published:** 2020-09-30

**Authors:** Aleš Štefančič, Samuel J. R. Holt, Martin R. Lees, Clemens Ritter, Matthias J. Gutmann, Tom Lancaster, Geetha Balakrishnan

**Affiliations:** 1 University of Warwick, Department of Physics, Coventry, CV4 7AL United Kingdom; 2 Institut Laue Langevin, 38042 Grenoble Cedex, France; 3 ISIS Facility, Rutherford Appleton Laboratory, Oxfordshire, OX11 0QX United Kingdom; 4 Durham University, Department of Physics, South Road, Durham, DH1 3LE United Kingdom

Correction to: *Scientific Reports* 10.1038/s41598-020-65676-9, published online 17 June 2020

The original version of this Article contained typographical errors.

In the Abstract,

“Each of these materials crystallizes in the *F*$\stackrel{-}{4}\stackrel{-}{4}$3*m* space group at ambient temperature.”

now reads:

“Each of these materials crystallizes in the *F*$\stackrel{-}{4}$3*m* space group at ambient temperature.”

In addition, in the figure legend for Figure 1,

“The V_4_S_2.85_/Se_1.15_ heterocubane (top right) and GaS_1.51_Se_2.49_ tetrahedra (bottom right), and their NaCl-like arrangement in the crystal structure (left side).”

now reads:

“The V_4_S_2.85_Se_1.15_ heterocubane (top right) and GaS_1.51_Se_2.49_ tetrahedra (bottom right), and their NaCl-like arrangement in the crystal structure (left side).”

Finally, in the Author Contribution Statement,

“A.S. and S.J.R.H. carried out the characterisation measurements with M.R.L. and analysed the data with M.R.L.”

now reads:

“A.Š. and S.J.R.H. carried out the characterisation measurements with M.R.L. and analysed the data with M.R.L.”

These errors have now been corrected in the PDF and HTML versions of the Article.

