# Supplementary material for: Establishing magneto-structural relationships in the solid solutions of the skyrmion hosting family of materials: GaV4S8−ySey
Source: Sci Rep. 2020 Jun 17;10:9813. doi: 10.1038/s41598-020-65676-9 (PMC7299962; doi:10.1038/s41598-020-65676-9)
Supplement: Supplementary file 1 — Supplementary Information. [file 41598_2020_65676_MOESM1_ESM.pdf]

Establishing magneto-structural relationships in the solid solutions of the skyrmion hosting family of materials: $GaV_4S_{8-y}Se_y$

Aleš Štefančič[1,*], Samuel J. R. Holt[1], Martin R. Lees[1], Clemens Ritter[2], Matthias J. Gutmann[3], Tom Lancaster[4], and Geetha Balakrishnan[1,+]

[1]University of Warwick, Department of Physics, Coventry, CV4 7AL, United Kingdom
[2]Institut Laue Langevin, 38042 Grenoble Cedex, France
[3]ISIS Facility, Rutherford Appleton Laboratory, Oxfordshire, OX11 0QX, United Kingdom
[4]Durham University, Department of Physics, South Road, Durham DH1 3LE, United Kingdom
[*]A.Stefancic@warwick.ac.uk
[+]G.Balakrishnan@warwick.ac.uk

ABSTRACT

The $GaV_4S_{8-y}Se_y$ ($y = 0$ to 8) family of materials have been synthesized in both polycrystalline and single crystal form, and their structural and magnetic properties thoroughly investigated. Each of these materials crystallizes in the $F\bar{4}3m$ space group at ambient temperature. However, in contrast to the end members GaV_4S_8 and GaV_4Se_8, that undergo a structural transition to the $R3m$ space group at 42 and 41 K respectively, the solid solutions ($y = 1$ to 7) retain cubic symmetry down to 1.5 K. In zero applied field the end members of the family order ferromagnetically at 13 K (GaV_4S_8) and 18 K (GaV_4Se_8), while the intermediate compounds exhibit a spin-glass-like ground state. We demonstrate that the magnetic structure of GaV_4S_8 shows localization of spins on the V cations, indicating that a charge ordering mechanism drives the structural phase transition. We conclude that the observation of both structural and ferromagnetic transitions in the end members of the series in zero field is a prerequisite for the stabilization of a skyrmion phase, and discuss how the absence of these transitions in the $y = 1$ to 7 materials can be explained by their structural properties.

Table 1. Refined parameters of $GaV_4S_{8-y}Se_y$ $(y = 0 \text{ to } 8)$ in the $F\bar{4}3m$ space group at room temperature from powder X-ray diffraction data taken on a laboratory diffractometer.

y	0	1	2	3	4	5	6	7	8
a Å	9.66294(7)	9.71632(6)	9.78401(4)	9.85542(9)	9.92004(6)	9.98498(5)	9.9800(2)<br>10.03670(16)	10.09205(7)	10.14102(3)
V (Å^3)	902.25(2)	917.288(16)	936.593(12)	957.25(2)	976.203(17)	995.500(16)	994.02(7)<br>1011.05(5)	1027.87(2)	1042.905(8)
sof S1	1	0.910(10)	0.719(11)	0.574(8)	0.413(9)	0.176(10)	0.06(4)<br>0.096(18)	0.05	N/A
sof S2	1	0.938(11)	0.882(10)	0.798(9)	0.692(8)	0.497(10)	0.55(5)<br>0.42(2)	0.230(14)	N/A
sof Se1	N/A	0.090(10)	0.281(11)	0.426(8)	0.587(9)	0.824(10)	0.94(4)<br>0.904(18)	0.95	1
sof Se2	N/A	0.062(11)	0.118(10)	0.202(9)	0.308(8)	0.503(10)	0.45(5)<br>0.58(2)	0.770(14)	1
Formula based on sof	GaV_4S_8	$GaV_4S_{7.39}Se_{0.61}$	$GaV_4S_{6.40}Se_{1.60}$	$GaV_4S_{5.49}Se_{2.51}$	$GaV_4S_{4.42}Se_{3.58}$	$GaV4S_{2.69}Se_{5.31}$	$GaV4S_{2.44}Se_{5.56}$<br>23.3(9)%<br>$GaV_4S_{2.06}Se_{5.94}$<br>76.7(9)%	$GaV_4S_{1.12}Se_{6.88}$	GaV_4Se_8
Formula weight (g/mol)	530.01	558.62	605.04	647.72	697.89	779.02	790.75<br>808.75	847.49	905.17
Phase purity	Phase pure	Phase pure	Phase pure	Phase pure	Phase pure	Phase pure	Two phases	VSe_2 impurity 3.3%	VSe_2 impurity 2.0%
R_{wp}	17.07	16.95	16.99	18.08	17.65	17.77	26.81	31.10*	23.21*
R_{exp}	14.90	13.90	13.32	14.12	13.47	12.48	19.40	9.47	12.50
χ^2	1.31	1.49	1.63	1.64	1.72	2.03	1.91	10.79*	3.45*
Atomic positions									
Ga on 4a	0	0	0	0	0	0	0<br>0	0	0
V on 16e (x, x, x)	0.60508(14)	0.60516(14)	0.60487(14)	0.60442(16)	0.60336(18)	0.60327(18)	0.6042(8)<br>0.6034(3)	0.6049(4)	0.6001(3)
S1 on 16e (x, x, x)	0.3709(2)	0.3730(7)	0.3722(7)	0.369(4)	0.368(6)	0.3790(15)	0.37(3)<br>0.374(6)	0.398(2)	N/A
S2 on 16e (x, x, x)	0.8631(2)	0.8634(2)	0.86460(19)	0.86454(19)	0.86399(18)	0.86237(16)	0.8656(6)<br>0.8649(3)	0.8637(3)	N/A
Se1 on 16e (x, x, x)	N/A	0.359(4)	0.360(2)	0.367(7)	0.369(6)	0.3671(5)	0.366(16)<br>0.3671(18)	0.3685(4)	0.36869(18)
Se2 on 16e (x, x, x)	N/A	0.8634(2)	0.86460(19)	0.86454(19)	0.86399(18)	0.86237(16)	0.8656(6)<br>0.8649(3)	0.8637(3)	0.85940(18)

* Higher R_{wp} and χ^2 values are due to the presence of the preferred orientation in VSe_2 (trigonal, $P\bar{3}m1$).

Table 2. Crystallographic data of $GaV_4S_{8-y}Se_y$ $(y = 0 \text{ to } 8)$ single crystals obtained from single crystal X-ray diffraction performed on a laboratory diffractometer.

y	0	1	2	3	4	5	6	7	8
a (Å)	9.64873(6)	9.67159(11)	9.73879(9)	9.80152(13)	9.88086(12)	9.96041(15)	10.01498(9)	10.06360(10)	10.12510(10)
V (Å^3)	898.277(18)	904.68(3)	923.67(3)	941.63(4)	964.68(4)	988.17(5)	1004.50(3)	1019.20(3)	1038.00(3)
Z	4	4	4	4	4	4	4	4	4
sof S1	1	0.969(3)	0.904(3)	0.822(4)	0.713(5)	0.553(7)	0.407(8)	0.255(8)	N/A
sof S2	1	0.934(4)	0.783(3)	0.599(4)	0.377(6)	0.237(8)	0.143(8)	0.047(8)	N/A
sof Se1	N/A	0.031(3)	0.097(3)	0.178(4)	0.287(5)	0.447(7)	0.593(8)	0.745(8)	1
sof Se2	N/A	0.066(4)	0.217(3)	0.401(4)	0.623(6)	0.763(8)	0.857(8)	0.953(8)	1
Empirical formula	S_8	$S_{7.61}Se_{0.39}$	$S_{6.74}Se_{1.25}$	$S_{5.68}Se_{2.32}$	$S_{4.20}Se_{3.80}$	$S_{3.12}Se_{4.88}$	$S_{2.20}Se_{5.80}$	$S_{1.09}Se_{6.91}$	Se_8
M_R (g/mol)	529.6	548.13	588.82	638.53	700.62	756.6	801.92	849.11	905.09
ρ_{calc} (g/cm^3)	3.916	4.024	4.234	4.504	4.824	5.086	5.303	5.534	5.792
Crystal size (mm3)	0.137 × 0.116 × 0.09	0.075 × 0.074 × 0.052	0.087 × 0.064 × 0.061	0.105 × 0.087 × 0.059	0.146 × 0.126 × 0.098	0.078 × 0.07 × 0.069	0.134 × 0.117 × 0.078	0.148 × 0.101 × 0.097	0.124 × 0.101 × 0.055
μ/mm^{-1}	8.75	10.161	13.174	16.785	21.097	24.736	27.639	30.853	34.019
Absor. correction	Analytical	Analytical	Analytical	Analytical	Analytical	Analytical	Analytical	Analytical	Analytical
T_{min}	0.445	0.472	0.429	0.308	0.131	0.231	0.103	0.066	0.097
T_{max}	0.558	0.576	0.517	0.427	0.264	0.314	0.247	0.192	0.271
2θ range (°)	7.314 to 89.284	7.298 to 90.1	7.248 to 89.464	7.2 to 89.794	7.142 to 90.066	7.086 to 89.882	7.048 to 89.838	7.014 to 89.86	6.97 to 89.732
reflection collected	17161	1596	2872	2447	3336	2380	6250	16197	5452
indep. Reflect.	420	420	430	438	441	454	470	472	481
restrains/ paramet.	0/12; 0/13	0/16	0/17	0/17; 0/18	0/17	0/18	0/17	0/17	0/12; 0/13
R_{int}	0.0493	0.0191	0.0151	0.0151	0.0189	0.0182	0.0358	0.0603	0.0343
R_1, wR_2 $[I \geq 2\delta\ (I)]$	0.0118, 0.0293	0.0143, 0.0326	0.0100, 0.0249	0.0120, 0.0298	0.0115, 0.0318	0.0154, 0.0387	0.0146, 0.0387	0.0142, 0.0361	0.0143, 0.0369
Goodness-of-fit on F^2	1.251	1.069	1.137	1.169	1.262	1.158	1.143	1.192	1.2
Largest diff. peak/hole $\left(e\text{Å}^{-3}\right)$	0.50/ -0.68	0.34/ -0.45	0.30/ -0.44	0.37/ -0.57	0.54/ -0.74	0.47/ -0.67	0.62/ -0.42	0.53/ -0.59	1.17/ -0.92
Flack paramet.	0.010(10); N/A	-0.021(18)	0.012(9)	-0.011(15); N/A	0.011(14)	N/A	-0.011(15)	0.007(15)	0.003(10); N/A
twin ratio	N/A; 0.984(12)	N/A	N/A	N/A; 1.006(19)	N/A	0.14(2)	N/A	N/A	N/A; 0.993(19)

Table 3. Crystallographic data for $GaV_4S_{8-y}Se_y$ $(y = 0$ to $8)$ single crystals along with selected bond distances, angles and tetrahedrons volumes from single crystal X-ray diffraction performed on a laboratory diffractometer. Bond distances, angles, and tetrahedral volumes are given in formats X-Y, X-Y-Z, and X_4 tetrahedra, respectively.

y	0	1	2	3	4	5	6	7	8
					Atomic positions				
Ga on 4a	0	0	0	0	0	0	0	0	0
V on 16e (x, x, x)	0.39407(2); 0.60593(2)	0.60576(2)	0.60529(2)	0.39527(2); 0.60473(2)	0.60408(2)	0.60393(3)	0.60389(3)	0.60397(4)	0.39553(3); 0.60447(3)
S1 on 16e (x, x, x)	0.62949(3); 0.37051(3)	0.37072(18)	0.37166(17)	0.6279(3); 0.3721(3)	0.3737(3)	0.3745(7)	0.3751(7)	0.3756(13)	N/A
S2 on 16e (x, x, x)	0.13590(3); 0.86410(3)	0.86418(4)	0.86426(2)	0.13584(2); 0.86416(2)	0.86387(2)	0.86367(3)	0.86349(3)	0.86336(2)	N/A
Se1 on 16e (x, x, x)	N/A	0.364(2)	0.3629(6)	0.6354(5); 0.3646(5)	0.3647(3)	0.3659(3)	0.36664(19)	0.36741(18)	0.63174(2); 0.36826(2)
Se2 on 16e (x, x, x)	N/A	0.86418(4)	0.86426(2)	0.13584(2); 0.86416(2)	0.86387(2)	0.86367(3)	0.86349(3)	0.86336(2)	0.13668(2); 0.86332(2)
				Selected bond distances in Å, angles in ° and tetrahedral volumes in Å^3					
V - S1	2.2941(4)	2.2959(18)	2.2973(17)	2.303(3)	2.297(3)	2.305(7)	2.311(7)	2.317(13)	N/A
V - Se1	N/A	2.375(19)	2.401(6)	2.392(5)	2.405(3)	2.409(3)	2.4124 (19)	2.4152(19)	2.4233(4)
V-V (intra)	2.8909(4)	2.8931(4)	2.9003(4)	2.9034(3)	2.9088(3)	2.9279 (6)	2.9429(6)	2.9594(7)	2.9918(5)
V-S1-V	78.11(1)	78.11(6)	78.28(5)	78.17(9)	78.55(9)	78.8(2)	79.1(2)	79.4(4)	N/A
V-Se1-V	N/A	75.1(6)	74.3(2)	74.74(14)	74.41(9)	74.86(9)	75.17(6)	75.56(6)	76.24(1)
S1-V-Se1	N/A	2.0(5)	2.57(15)	2.22(14)	2.68(10)	2.59(19)	2.56(18)	2.5(3)	N/A
V-V (inter)	3.9318(3)	3.9457(4)	3.9861(4)	4.0276(5)	4.0786(5)	4.1151(6)	4.1388(6)	4.1566 (7)	4.1677(6)
V_4 tetrahedra	2.8473(9)	2.8538(9)	2.8750(10)	2.8844(10)	2.9004(10)	2.9582(15)	3.0036(15)	3.055(2)	3.1560(16)
$S1_4$ tetrahedra	5.201(2)	5.213(13)	5.207(12)	5.25(2)	5.18(2)	5.21(5)	5.22(5)	5.23(10)	N/A
$Se1_4$ tetrahedra	N/A	6.07(15)	6.35(5)	6.23(4)	6.37(2)	6.35(3)	6.353(16)	6.335(15)	6.3288(17)
V_4cent-V_4cent	6.82268(3)	6.83885(6)	6.88636(5)	6.93072(7)	6.98682(6)	7.04307(8)	7.08166(5)	7.11604(5)	7.15953(5)
V-S2/Se2	2.5244(4)	2.5329(4)	2.5567(3)	2.5791(3)	2.6057(3)	2.6271(4)	2.6406(4)	2.6515(5)	2.6612(4)
Ga-S2/Se2	2.2712(3)	2.2752(4)	2.2897(2)	2.3061(2)	2.3297(2)	2.3520(3)	2.3680(3)	2.3817(2)	2.3970(2)
Ga-S2/Se2-V	115.941(13)	115.923(16)	115.823(9)	115.639 (9)	115.368 (9)	115.260(13)	115.188 (13)	115.165 (12)	115.284(10)
Ga-V	4.0678(2)	4.0781(2)	4.1084(2)	4.1373(2)	4.1736(2)	4.2079(3)	4.2311(3)	4.2513(4)	4.2750(3)
Ga-Ga	6.82268(3)	6.83885(6)	6.88636(5)	6.93072(7)	6.98682(6)	7.04307(8)	7.08166(5)	7.11604(5)	7.15953(5)
S2/Se2$_4$ tetrahedra	6.012(2)	6.044(3)	6.1604(16)	6.2941(16)	6.4895 (17)	6.677(3)	6.814(3)	6.9337(18)	7.0678(18)

Table 4. Powder neutron diffraction refined parameters of GaV_4S_8 on D2B diffractometer at the ILL.

Temperature (K)	50		30	15	9	1.5
Space Group	$F\bar{4}3m$		$R3m$	$R3m$	$R3m$	$R3m$
Z	4		3	3	3	3
a Å	9.64823(5)		6.80183(6)	6.80101(6)	6.80098(6)	6.80084(6)
c Å	N/A		16.7984(3)	16.8034(3)	16.8058(3)	16.8053(3)
V (Å^3)	898.139(13)		673.055(18)	673.091(18)	673.182(17)	673.133(18)
R_{wp}	4.79		4.61	4.62	4.52	4.53
R_{exp}	3.77		3.77	3.77	3.77	3.77
χ^2	1.62		1.49	1.50	1.44	1.44
			Atomic positions			
Ga on 4a	0	Ga on 3a (z fixed)	0	0	0	0
S1 on 16e (x, x, x)	0.37023(12)	S1 on 3a (0, 0, z)	0.6312(5)	0.6314(4)	0.6308(4)	0.6312(4)
S2 on 16e (x, x, x)	0.86405(11)	S2 on 9b (x, 2x, z)	0.1719(3) 0.4551(3)	0.1716(3) 0.4549(3)	0.1716(3) 0.4547(3)	0.1716(3) 0.4548(3)
		S3 on 9b (x, 2x, z)	0.1802(3) 0.9525(3)	0.1796(3) 0.9520(3)	0.1797(3) 0.9522(3)	0.1800(3) 0.9523 (3)
		S4 on 3a (0, 0, z)	0.1347(4)	0.1353(4)	0.1357(4)	0.1356(4)

Table 5. Single crystal neutron diffraction refined parameters of $GaV_4S_4Se_4$ from data collected on the SXD diffractometer at ISIS. Positions of the V atoms are constrained to those from the single crystal X-ray diffraction.

Temperature (K)	55	1.5
Space Group	$F\bar{4}3m$	$F\bar{4}3m$
Z	4	4
a Å	9.89110(10)	9.89250(10)
V (Å^3)	967.684(17)	968.095(17)
M_R (g/mol)	700.7	700.7
ρ_{calc} (g/cm^3)	4.8094	4.8074
Crystal size (mm^3)	$1 \times 1.5 \times 1.5$	$1 \times 1.5 \times 1.5$
2θ range (°)	3.61 to 73.22	3.56 to 73.25
reflection collected	512	1798
indep. Reflect.	509	1784
constraints/ paramet.	5/12	5/12
R_{int}		
R_1, wR_2 $[I \geq 2\delta\ (I)]$	0.0804, 0.1610	0.0804, 0.1506
Goodness-of-fit on F^2	2.03	2.65
Atomic positions		
Ga on 4a	0	0
V on 16e (x, x, x)	0.60408	0.60408
S1 on 16e (x, x, x)	0.8638(2)	0.86401(8)
Se1 on 16e (x, x, x)	0.8638(2)	0.86401(8)
S2 on 16e (x, x, x)	0.86332(5)	0.3717(5)
Se2 on 16e (x, x, x)	0.3740(11)	0.3656(5)

Table 6. Single crystal neutron diffraction refined parameters of GaV_4Se_8 on SXD diffractometer at the ISIS.

Temperature (K)	55		30
Space Group	$F\bar{4}3m$		$R3m$
Z	4		3
a Å	10.11750(10)		7.1490(18)
c Å	N/A		17.524(6)
V (Å^3)	1035.666(18)		775.6(4)
M_R (g/mol)	905.2		905.2
ρ_{calc} (g/cm^3)	5.8052		5.8135
Crystal size (mm^3)	$2 \times 2 \times 2$		$2 \times 2 \times 2$
2θ range (°)	3.44 to 82.14		3.44 to 78.84
reflection collected	4740		3637
indep. Reflect.	2948		2638
constraints/ paramet.	0/15		8/16
R_{int}			
R_1, wR_2 $[I \geq 2\delta\ (I)]$	0.0701, 0.1426		0.0652, 0.1276
Goodness-of-fit on F^2	1.40		1.52
Atomic positions			
Ga on 4a	0	Ga on 3a (z fixed)	0
S1 on 16e (x, x, x)	0.36822(5)	S1 on 3a (0, 0, z)	0.63170(14)
S2 on 16e (x, x, x)	0.86332(5)	S2 on 9b (x, 2x, z)	0.17595(9) 0.45560(12)
		S3 on 9b (x, 2x, z)	0.18184(10) 0.95444(12)
		S4 on 3a (0, 0, z)	0.13636(14)

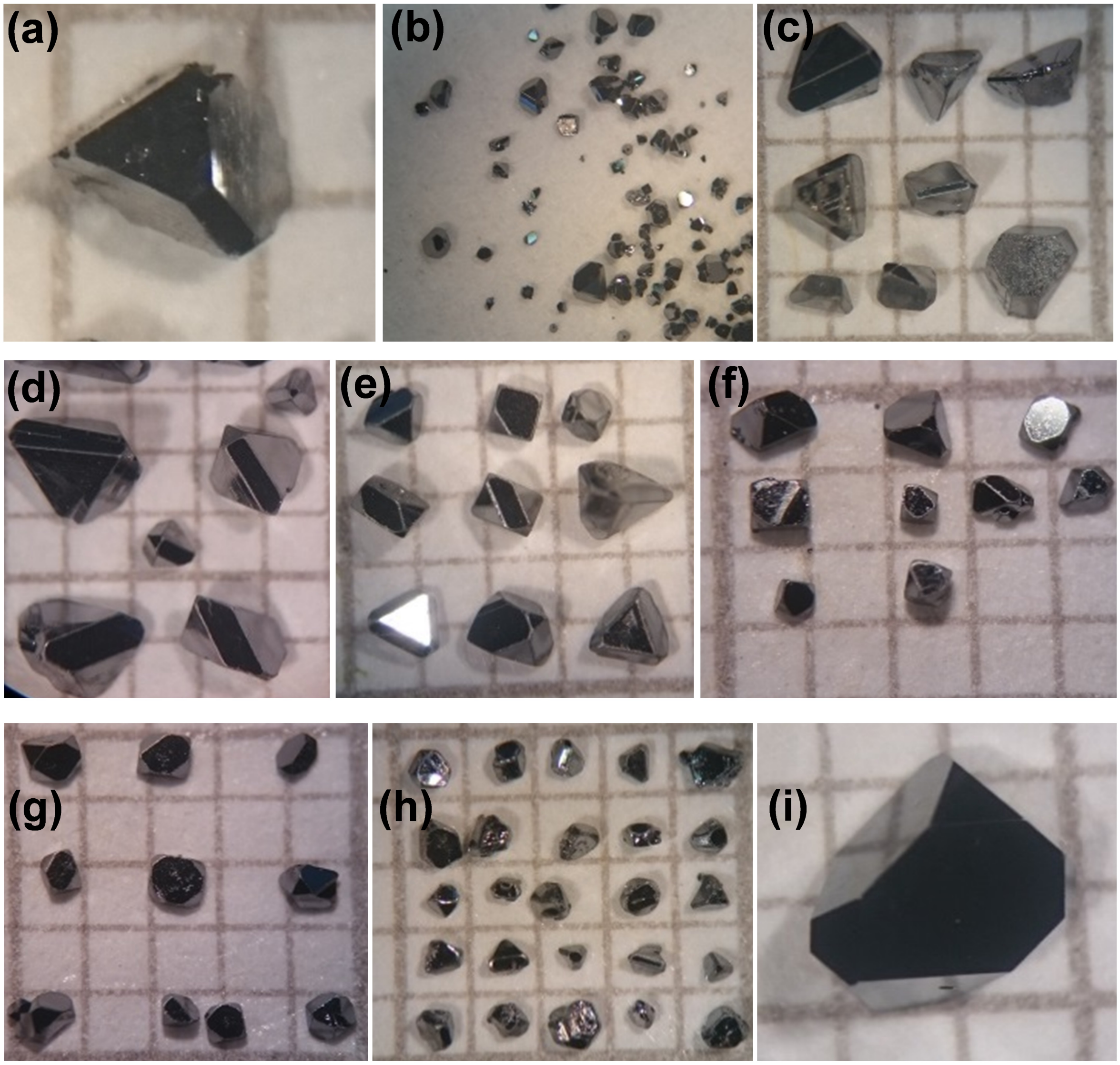

Figure 1. Crystals of $GaV_4S_{8-y}Se_y$ (a) $y = 0$, (b) $y = 1$, (c) $y = 2$, (d) $y = 3$, (e) $y = 4$, (f) $y = 5$, (g) $y = 6$, (h) $y = 7$, and (i) $y = 8$ photographed on millimetre paper.

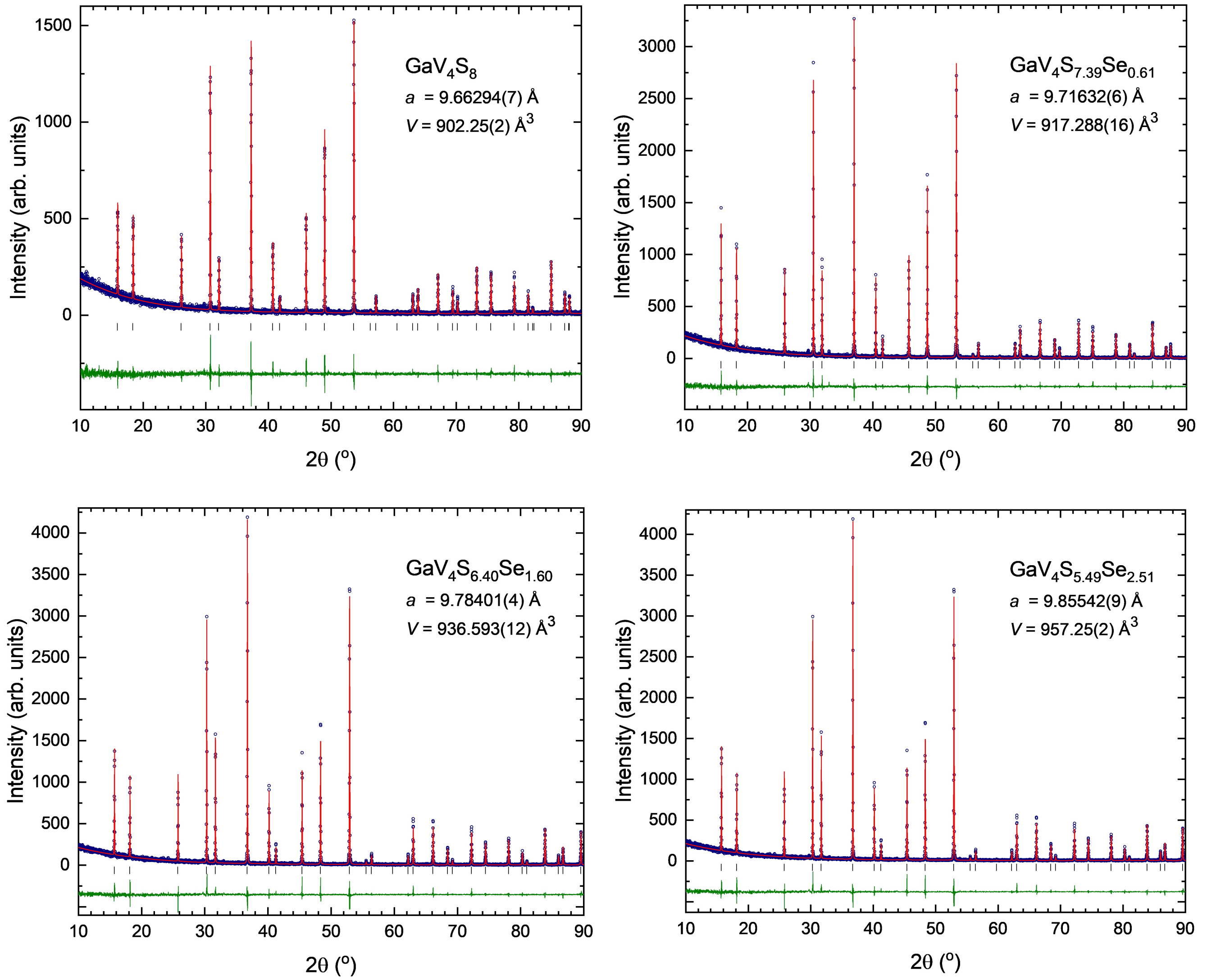

Figure 2. Powder X-ray diffraction profiles of GaV_4S_8, GaV_4S_7Se, $GaV_4S_6Se_2$, and $GaV_4S_5Se_3$. The experimentally-obtained diffraction profile at ambient temperature (blue open circles), refinement based on the model obtained from single crystal X-ray diffraction at room temperature (red solid line), difference (olive green solid line) and predicted peak positions (black tick marks).

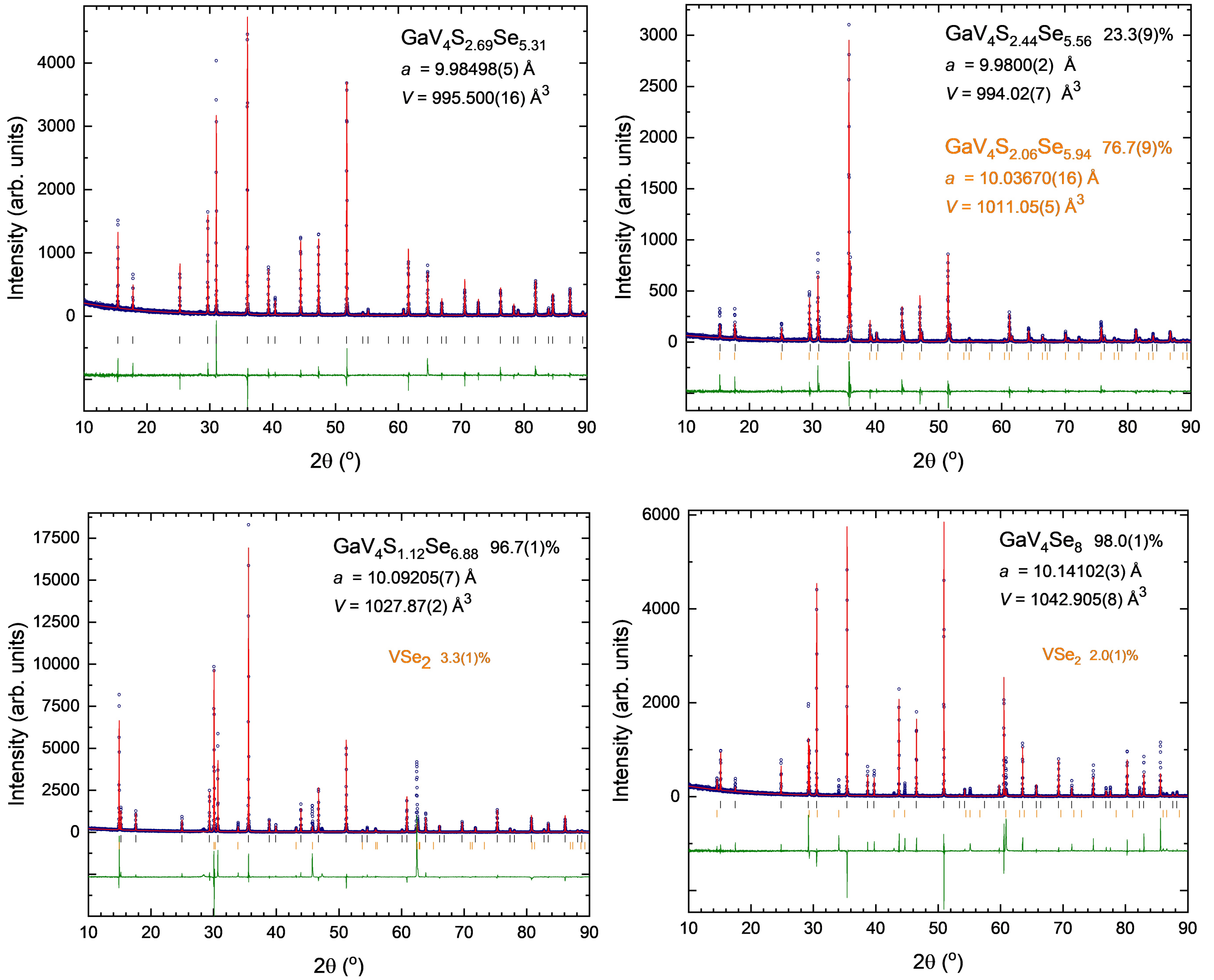

Figure 3. Powder X-ray diffraction profiles of $GaV_4S_3Se_5$, $GaV_4S_2S_6$, GaV_4SSe_7, and GaV_4Se_8. The experimentally-obtained diffraction profile at ambient temperature (blue open circles), refinement based on the model obtained from single crystal X-ray diffraction at room temperature (red solid line), difference (olive green solid line) and predicted peak positions (black tick marks).

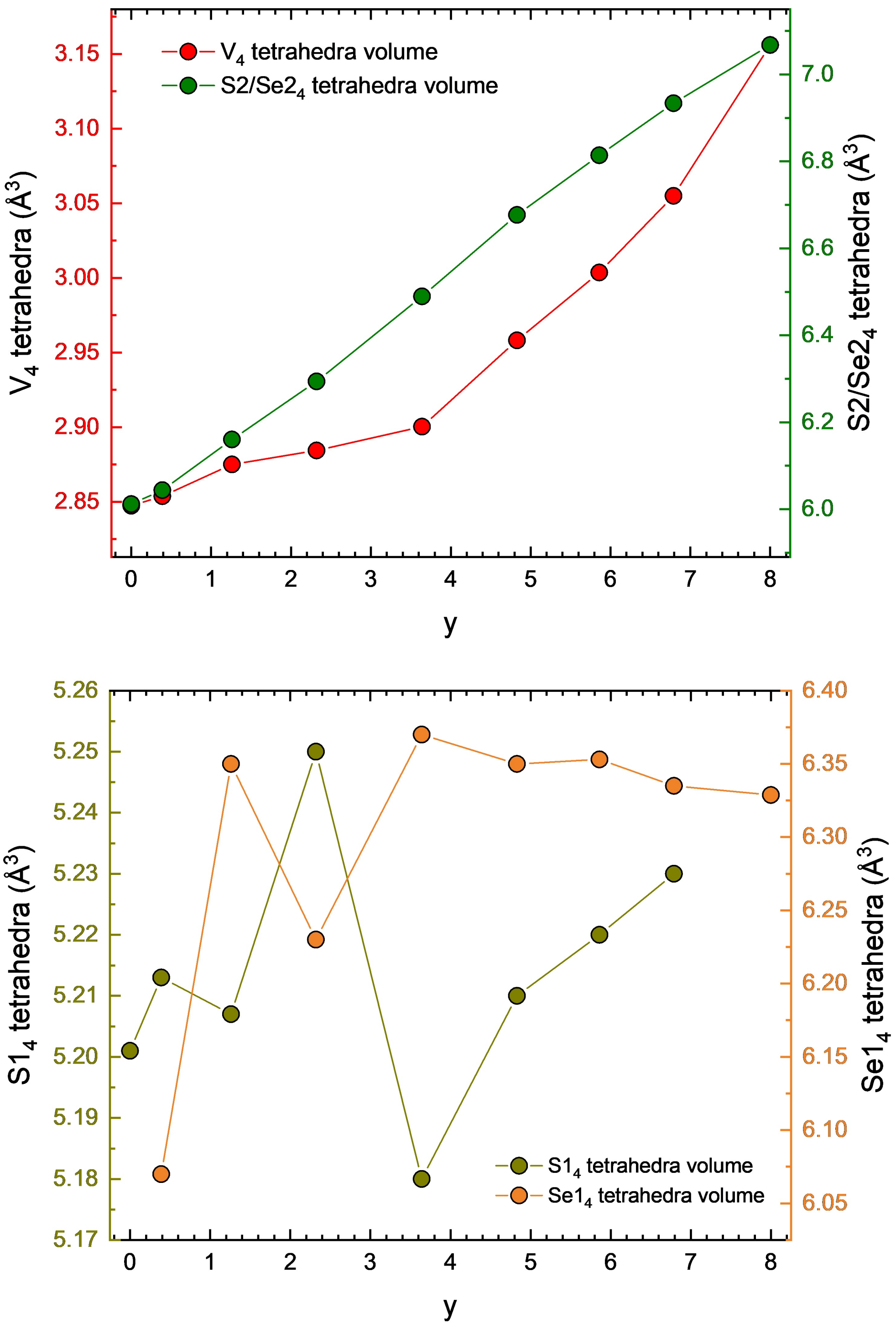

Figure 4. Top: volume of the V_4 tetrahedra and the S/Se site 2 tetrahedra. Bottom: volume of the S and Se tetrahedra within the V cluster for different compositions of $GaV_4S_{8-y}Se_y$.

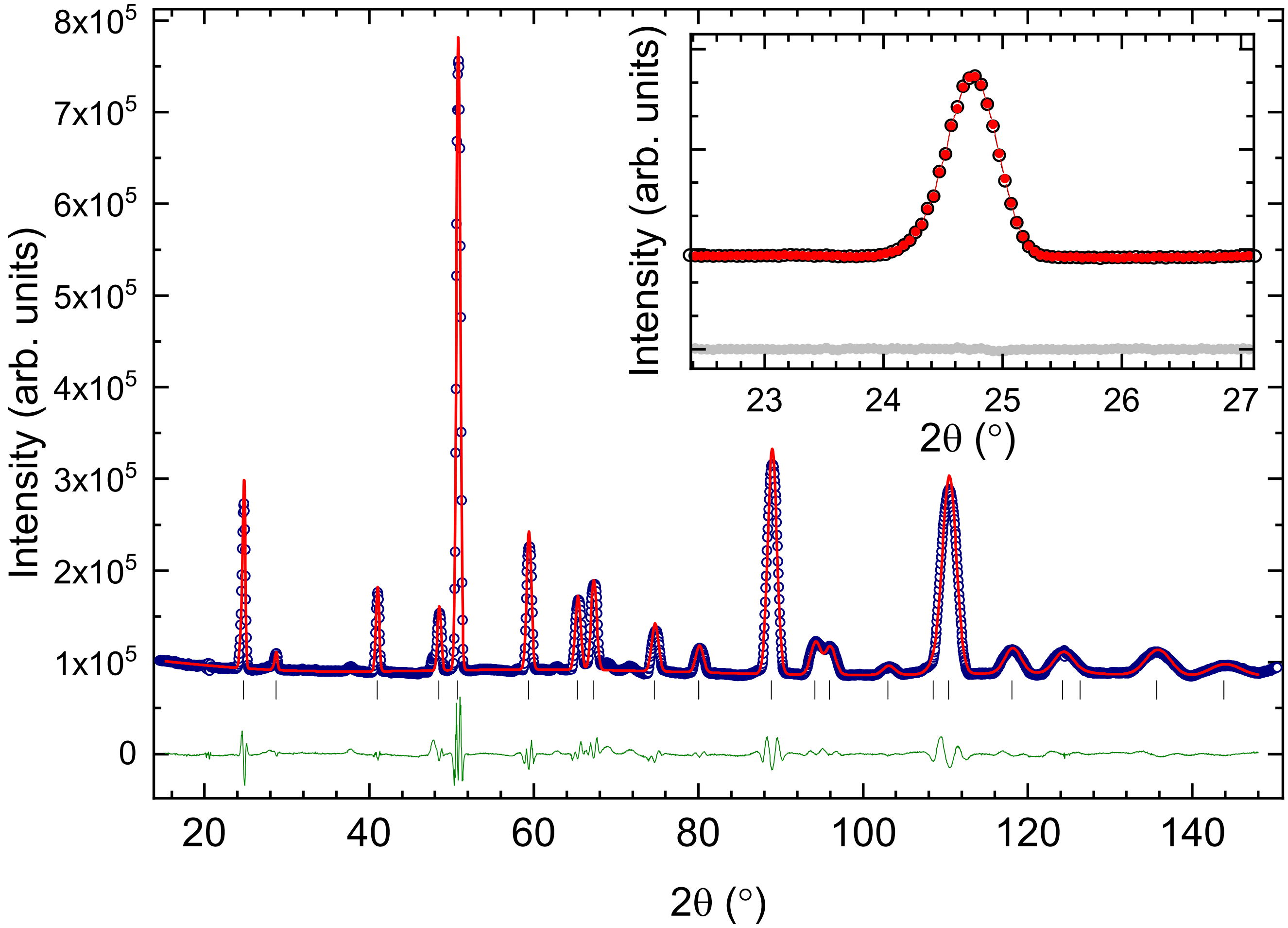

Figure 5. $GaV_4S_6Se_2$ powder neutron diffraction profile taken on the D20 diffratometer at the ILL. The experimentally-obtained diffraction profile at 15 K (blue open circles), refinement based on the model obtained from single crystal X-ray diffraction at room temperature (red solid line), difference (olive green solid line) and predicted peak positions (black tick marks). The inset shows the (111) diffraction peak taken at 15 K (black open circles) and 1.5 K (red closed circles) and difference (grey closed circles). At 1.5 and 15 K the lattice parameter refine in an $F\bar{4}3m$ to be 9.7673(4) and 9.7669(4) respectively.

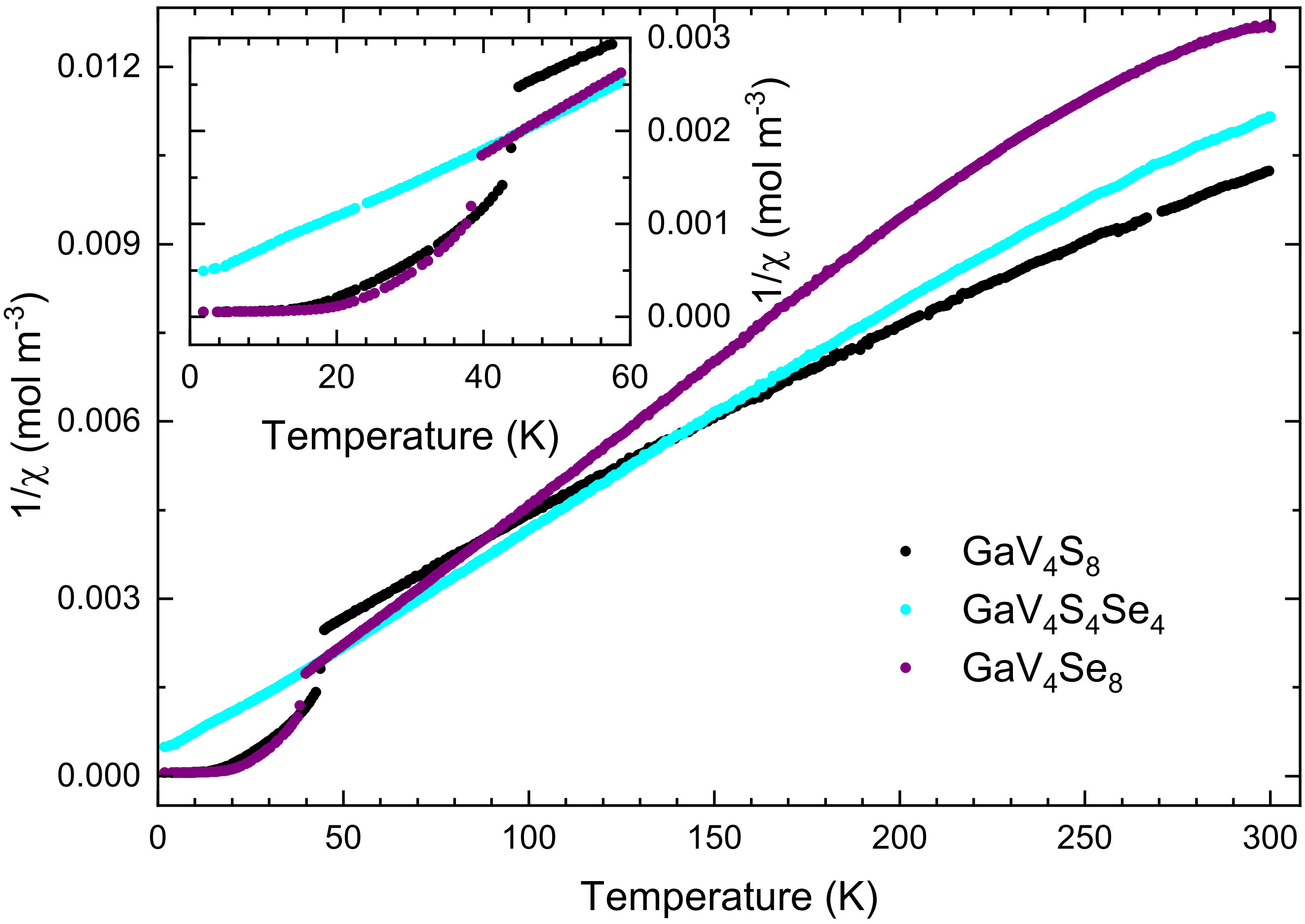

Figure 6. Inverse magnetic susceptibility vs temperature for GaV_4S_8, $GaV_4S_4Se_4$, and GaV_4Se_8 measured in an applied magnetic field of 10 mT.
